# Associations of Leukocyte Telomere Length With Aerobic and Muscular Fitness in Young Adults

**DOI:** 10.1093/aje/kww123

**Published:** 2017-03-01

**Authors:** Dylan M. Williams, Jessica L. Buxton, Marko T. Kantomaa, Tuija H. Tammelin, Alexandra I. F. Blakemore, Marjo-Riitta Järvelin

**Keywords:** aerobic fitness, biological aging, handgrip strength, Northern Finland Birth Cohorts, telomere length

## Abstract

Decline in both telomere length and physical fitness over the life course may contribute to increased risk of several chronic diseases. The relationship between telomere length and aerobic and muscular fitness is not well characterized. We examined whether there are cross-sectional associations of mean relative leukocyte telomere length (LTL) with objective measures of aerobic fitness, muscle strength, and muscle endurance, using data on 31-year-old participants of the Northern Finland Birth Cohort 1966 (*n* = 4,952–5,205, varying by exposure-outcome analysis). Aerobic fitness was assessed by means of heart rate measurement following a standardized submaximal step test; muscular fitness was assessed by means of a maximal isometric handgrip strength test and a test of lower-back trunk muscle endurance. Longer LTL was associated with higher aerobic fitness and better trunk muscle endurance in models including adjustment for age, sex, body mass index, socioeconomic position, diet, smoking, alcohol consumption, physical activity level, and C-reactive protein. In a sex-stratified analysis, LTL was not associated with handgrip strength in either men or women. LTL may relate to aspects of physical fitness in young adulthood, but replication of these findings is required, along with further studies to help assess directions and causality in these associations.

Reduced aerobic and muscular fitness are hallmarks of the aging process and manifest as physical inactivity, frailty, and sarcopenia (muscle wastage) in old age (1, 2). Declining fitness may raise the risks of both morbidity and mortality: Poor fitness is associated with increased risk of a number of age-related chronic diseases and all-cause mortality (3, 4). Currently, the biological mechanisms underlying the decline in aerobic and muscular fitness with age are incompletely understood. One factor that may be related to both long-term cardiorespiratory health and muscle performance is telomere length (TL).

Telomeres are heterochromatin structures formed of repetitive DNA sequences and specialized proteins which cap the ends of chromosomes (5). Telomeres are progressively eroded during successive rounds of mitosis, with additional attrition attributed to oxidative stress and inflammation (6, 7). There is wide variation in TL between individuals of equal chronological age, due to differences in both genetic factors and environmental determinants (8–10). When telomeres reach a critically short threshold, they can trigger cellular senescence or apoptosis, along with associated metabolic changes, suggesting a potential mechanism by which shortened telomeres could raise disease risk with increasing age (7). Shorter age-adjusted mean leukocyte telomere length (LTL) is associated with decreased longevity and a range of chronic diseases (5, 11, 12), and TL may be causally related to some of these (8). However, it is unclear what role (if any) TL plays in the decline in physical fitness with age, whether long-term variation in physical activity modifies TL over time, or whether there is a bidirectional relationship between these factors.

Several cross-sectional and prospective studies have examined objective measures of physical fitness in relation to either LTL or TL from skeletal muscle. A positive association of TL with aerobic fitness has been reported (tested by either submaximal or maximal oxygen consumption) (13–15), along with inconsistent but largely null findings for associations with handgrip strength (16–24). However, most studies to date have had relatively small sample sizes (only 2 have had more than 1,000 study participants), many have used elderly participants or special groups (e.g., endurance athletes), and several have lacked multiple standardized measures of aerobic fitness and different aspects of muscle fitness.

The aim of this study was to test for cross-sectional associations of LTL with objective measures of aerobic fitness and muscle strength and endurance in a large sample of Finnish participants at 31 years of age. We hypothesized that shorter LTL would be associated with lower aerobic fitness, handgrip strength, and trunk muscle endurance and that any associations would be partly mediated by low-grade inflammation.

## METHODS

### Participants

**Figure 1.**
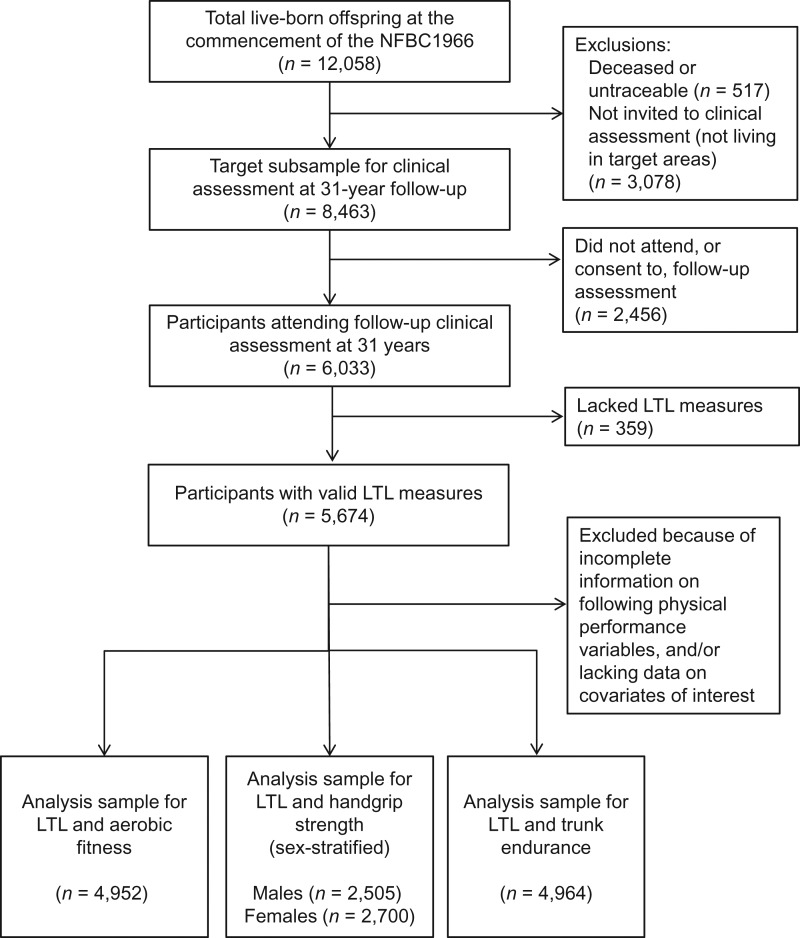
Derivation of the study sample, Northern Finland Birth Cohort 1966 (NFBC1966), 1966–1997. LTL, leukocyte telomere length.

The Northern Finland Birth Cohort 1966 is a prospective birth cohort study that aimed to recruit all pregnant women living in the provinces of Oulu and Lapland in northern Finland with expected delivery dates in 1966 (25). A total of 12,058 live-born offspring, all of white European ethnic origin, were enrolled in the cohort. Detailed information on the infants and their parents was collected starting prenatally, with further follow-up thereafter. In 1997, all living offspring (then aged 31 years) with known addresses were contacted and sent a postal questionnaire. A subset of 8,463 offspring residing in the areas of Oulu, Lapland, and Helsinki was also invited to undergo a clinical assessment, which included blood sampling. Of these participants, 6,033 attended the assessment. Informed written consent for the use of the data was obtained, and approval was granted by the Ethics Committee of the Northern Ostrobothnia Hospital District in Oulu, Finland, in accordance with the Declaration of Helsinki. For this study, analysis samples varied according to availability of physical fitness test data. The number of persons with valid information on LTL, fitness test measures, and all relevant covariates ranged from 4,952 to 5,205 (Figure 1).

### Telomere length

Blood samples drawn at the 31-year follow-up assessment in 1997 were collected between 8:00 am and 11:00 am, following an overnight fast by participants. Samples were stored at −70°C until thawed for analyses. Mean relative LTL was measured in genomic DNA samples prepared from peripheral blood leukocytes using a multiplex quantitative real-time polymerase chain reaction (qPCR) method (26), with modifications as described previously (27). Briefly, the multiplex qPCR method is based on a measure of the amplification of the telomeric DNA sequence (T) relative to that of a single copy gene (S) in each test sample, and normalized using a common reference DNA sample. This provides “T/S ratios” for each DNA sample, which are used as mean relative LTL values for participants. The overall mean coefficient of variation for T/S values of duplicate test samples on the same plate was 5%, and the mean interrun coefficient for randomly selected samples was 6.2%.

### Physical fitness measures

At the 31-year clinical assessment, 4 teams of research nurses supervised the measurements of aerobic and muscular fitness, performed anthropometric measurements, and carried out medical examinations (28). Before the fitness tests, participants were interviewed to exclude those with cardiovascular diseases or orthopedic problems. Fewer than 10% of participants were excluded from the analyses due to incomplete tests (most commonly, individuals did not undertake the step and/or trunk endurance tests because of acute ill health or pregnancy).

To measure submaximal aerobic fitness, participants undertook a 4-minute step test, making 23 ascents and descents onto a bench per minute in time to a metronome. Participants were shoeless during the test; bench heights were 33 cm for females and 40 cm for males. Heart rates were measured before and immediately after exercise using a heart rate monitor handle (Fitwatch by Polar Electro, Kempele, Finland), and these measures were used as an indicator of participants’ aerobic fitness—lower heart rates (beats/minute) after the step test indicating higher fitness (29).

Muscle strength was tested using measures of the maximal isometric handgrip strength (kg) of the dominant hand, using a hand dynamometer (Newtest, Oulu, Finland). Participants were standing with the hand beside the trunk but not touching it. Grip width was adjusted to hand size. The highest value from 3 attempts (lasting 2–4 seconds) was used as the test result.

Muscle endurance was assessed using a lower-back trunk muscle extension test (the Biering-Sorensen test) (30). Participants lay prone with their lower body lying on a stand and their upper body unsupported from the level of the anterior superior iliac spine upwards, with their arms beside their trunk and with their legs held down by a research nurse. Participants then held their upper torsos horizontally for as long as possible, for assessment of the isometric endurance capacity of trunk extensor muscles. Testing was stopped if the upper torso was no longer maintained horizontally or when 4 minutes had elapsed (regardless of whether or not the participant could continue), to avoid damaging the trunk muscles. Duration of the test performance (from 0 seconds to 240 seconds) was then used as a measure of endurance.

### Covariates

The following variables were regarded as potential confounders of associations of LTL with physical fitness variables: age, sex, adiposity (measured by body mass index (BMI)), socioeconomic position, diet quality, smoking, and alcohol consumption. Effect(s) of adjustment for physical activity on associations and potential mediation by C-reactive protein (CRP), a marker of low-grade inflammation, were also investigated.

BMI (weight (kg)/height (m)^2^) was derived from height and weight measured at the clinical assessment. BMI was derived from self-reported questionnaire responses if assessment measurements were unavailable (<0.2% of the sample).

Information on socioeconomic position, diet, smoking, alcohol intake, and physical activity was recorded from the 31-year follow-up postal questionnaire. Socioeconomic position was defined from responses on occupation and employment status, with the following categories: unskilled worker, skilled worker, professional, farmer, or other (retired, student, or long-term unemployed). A 6-category diet quality variable was calculated (0 = healthiest, 5 = unhealthiest) from frequencies of intake of several food groups in the prior 6 months (31). Alcohol consumption was calculated as a continuous variable (intake in g/day) from detailed questions on type, strength, and frequency of alcoholic drinks consumed. Leisure-time physical activity was calculated as metabolic equivalent of task hours per week, based on frequency and duration of both light and brisk physical activity (32). Three smoking status variables were derived from questions: never/ever smoking; current number of cigarettes smoked per day (continuous scale); and current user or nonuser of alternative tobacco products (cigars, pipes, snuff, or chewing tobacco).

Serum high-sensitivity CRP concentrations were determined by immunoenzymometric assay (Medix Biochemica, Espoo, Finland), using the same clinical samples as those used for LTL measurement. The intra- and interassay coefficients of variation were 4.2% and 5.2%, respectively.

### Statistics

In analyses of association, LTL was considered the exposure of interest and physical test measures were the outcome variables, which allowed more effective modeling of the trunk muscle endurance data and consistency of presentation across all models. However, there are plausible reasons for anticipating that any associations between LTL and physical fitness could represent a bidirectional relationship, particularly regarding aerobic fitness as a possible determinant of LTL (see commentary in the Discussion).

A natural logarithmic (log_n_) transformation was applied to LTL measurements (T/S ratios) to obtain an approximately normal distribution of this variable for use in all regression models. The log_n_-transformed LTL was *z* scored for models, so that results were expressed per 1-standard-deviation change in log_n_-transformed LTL. We used multivariable linear regression models to examine associations of LTL with aerobic fitness and handgrip strength and to adjust for potential confounders and mediators. The trunk muscle endurance distribution was right-censored because a large proportion of the analysis sample (27%) endured the full test duration. Thus, we modeled trunk muscle endurance as an outcome in a survival analysis using Cox proportional hazards regression, where hazard ratios reflected the risk of participants’ failing to complete the test at some point within 4 minutes.

We constructed several models investigating each potential exposure-outcome association. In model 1, we adjusted for age and sex. In model 2, we included further adjustment for potential confounding by BMI, socioeconomic position, diet quality, smoking, and alcohol consumption. Model 3 included the adjustments in model 2 plus CRP to examine potential mediation of associations by inflammation. Model 4 included the adjustments described for model 2 plus physical activity, to examine whether this affected the magnitude of any associations. A random-effects term (calculated by maximum likelihood estimation) was included in linear regression models to account for batch effects in LTL measurements. Robust standard errors were used in survival models. Handgrip strength has a bimodal distribution due to separate modal values for males and females, so we stratified all analyses of LTL and handgrip strength by sex. Therefore, adjustments in these models did not include sex.

Further to main analyses, we tested for interactions between sex and LTL in association with aerobic fitness and trunk endurance. Possible nonlinearity of associations of LTL with aerobic fitness and handgrip strength was tested by examining fractional polynomial statistics and examining graphical plots. Proportionality in Cox models was examined using Kaplan-Meier curves for 3 categories of the *z*-scored log_n_-LTL distribution. In case there were inaccuracies in the qPCR measurement of extreme LTL values, we repeated the main-model analyses after excluding a small number of participants with LTL-value outliers (≥3 standard deviations from the mean of the log_n_-transformed T/S ratio distribution; 48–51 persons were excluded, varying by LTL-outcome model). We also conducted additional analyses for a subsample of participants with complete information on aerobic fitness, handgrip strength, and trunk endurance (*n* = 4,733). In these analyses, we constructed models with adjustments identical to those of the main analyses, plus 1 further model with mutual adjustment for all physical fitness variables—that is, to examine the LTL–aerobic fitness association adjusted for handgrip strength and trunk endurance and to examine the LTL–trunk endurance association adjusted for aerobic fitness and handgrip strength.

## RESULTS

Table 1 shows the characteristics of the Northern Finland Birth Cohort 1966 participants included in the analyses, along with information on those excluded because of missing data. Generally, there were few notable differences between included and excluded participants; those excluded were on average slightly younger, had lower alcohol consumption, and had higher CRP levels and higher aerobic fitness (lower average heart rates indicating higher aerobic fitness).

**Table 1.** Characteristics of Northern Finland Birth Cohort 1966 Participants Included in Analyses of Telomere Length and Physical Fitness and Those Excluded Because of Missing Data on 1 or More Variables, 1966–1997

	Analysis Sample^a^			Excluded Participants (*n* ≤ 739)				*P* for Difference^b^
	%	Mean (SD)	Median (IQR)	No. Missing	%	Mean (SD)	Median (IQR)	
Age, years		31.2 (0.3)		634		31.1 (0.3)		0.001
Sex, % male	48.3			722	51.7			0.18
Body mass index^c^		24.7 (4.2)		690		24.7 (4.4)		0.97
Socioeconomic position				645				0.27
Farmer	3.8				2.3			
Professional	23.9				22.8			
Skilled worker	30.7				32.3			
Unskilled worker	25.7				25			
Other^d^	16				17.7			
Physical activity, MET-hours per week			11.3 (3.8–20.6)	662			10.3 (3.6–22.0)	0.47
Diet quality (lower score indicates a better diet)				669				0.74
0	7.5				6.7			
1	24.3				26			
2	30.2				28.7			
3	26.6				25.9			
4 or 5	11.5				12.7			
Tobacco use								
Ever smoking (vs. never)	63.4			633	63.2			0.93
Current smoking, cigarettes/day (*n* = 1,916)			10 (6–20)	229			12 (7–20)	0.75
Alternate tobacco products^e^	3.4				4.7			0.08
Alcohol consumption, g/day			4.2 (1.1–10.9)	526			3.5 (0.8–8.4)	0.002
C-reactive protein, mg/L			0.75 (0.36–1.87)	556			0.82 (0.38–2.22)	0.05
Leukocyte telomere length (T/S ratio^f^)			1.14 (0.92–1.42)	390			1.14 (0.92–1.41)	0.61
Step test heart rate, beats/minute		148.2 (17.3)		583			145.7 (17.5)	0.001
Handgrip strength, kg								
Men		49.6 (8.8)		279	49.5 (9.3)			0.88
Women		28.2 (6.3)		351	28.1 (6.1)			0.81
Trunk muscle endurance, seconds			170 (127–240)	585			172 (125–240)	0.92

Abbreviations: IQR, interquartile range; MET, metabolic equivalent of task; SD, standard deviation.

^a^ Characteristics of participants included in *any* of the 3 main analyses of associations between leukocyte telomere length and fitness variables (*n* ≤ 5,284).

^b^*P* for difference in characteristics between excluded and included participants, based on a 2-tailed *t* test, Kruskal-Wallis test, or χ^2^ test.

^c^ Weight (kg)/height (m)^2^.

^d^ Retired, student, long-term unemployed, or not defined.

^e^ Pipes, cigars, chewing tobacco, or snuff.

^f^ Amplification of the telomeric DNA sequence (T) relative to that of a single copy gene (S) in each test sample.

Web Table 1 (available at http://aje.oxfordjournals.org/) shows the characteristics of participants across 5 categories of the mean relative LTL distribution. There were decreasing trends in age, BMI, percentage of ever smokers, use of alternate tobacco products, alcohol consumption, CRP, and step-test heart rate across fifths of the LTL distribution (*P*’s for trend ≤ 0.06). Current smoking was also lower in the highest LTL fifth than in lower categories (*P* for trend = 0.10). There were increasing trends in percent female, diet quality, and trunk muscle endurance across fifths of the distribution (all *P*’s ≤ 0.02).

Results from multivariable linear regression examining the association of LTL with aerobic fitness are shown in Table 2. In model 1, which adjusted for age and sex, longer LTL was associated with higher aerobic fitness. This association was attenuated slightly, but remained, after adjustment for potential confounding by BMI, socioeconomic position, smoking status, diet quality, and alcohol consumption. BMI adjustment was responsible for most of this attenuation. There was a small degree of further attenuation with additional adjustment for CRP in model 3. Adjustment for physical activity in addition to other potential confounders (but not CRP) in model 4 produced results nearly identical to those from model 2.

**Table 2.** Associations of Leukocyte Telomere Length With Aerobic Fitness in Adults Aged 31 Years From Northern Finland Birth Cohort 1966 (*n* = 4,952), 1966–1997

Model	β^a^	95% CI	*P* Value
Model 1^b^	−0.7	−1.2, −0.2	0.004
Model 2^c^	−0.5	−1.0, −0.1	0.03
Model 3^d^	−0.5	−1.0, −0.01	0.05
Model 4^e^	−0.5	−1.0, −0.1	0.03

Abbreviations: CI, confidence interval; LTL, leukocyte telomere length.

^a^ Beta coefficients represent the average difference in heart rate (beats/minute) per 1-standard-deviation increase in mean relative LTL (lower heart rates indicating higher aerobic fitness).

^b^ Model 1—adjusted for age, sex, and LTL measurement batch.

^c^ Model 2—adjusted for model 1 variables plus body mass index, socioeconomic position, diet quality, smoking status, and alcohol consumption.

^d^ Model 3—adjusted for model 2 variables plus C-reactive protein.

^e^ Model 4—adjusted for model 2 variables plus physical activity.

Table 3 shows results from multivariable linear regression analyses examining associations of LTL with handgrip strength (stratified by sex) and results from Cox proportional hazards analyses of the association of LTL with trunk muscle endurance (for the full sample). There was no evidence of an association of LTL with handgrip strength in either men or women in any model. In contrast, longer LTL was associated with higher trunk muscle endurance (as evidenced by a lower hazard ratio for failing to complete the test with increasing LTL). This association remained after adjustment for all potential confounders, CRP, and physical activity (models 2–4).

**Table 3.** Associations of Leukocyte Telomere Length with Handgrip Strength (Stratified by Sex) and Trunk Muscle Endurance (Not Stratified) in Adults Aged 31 Years From Northern Finland Birth Cohort 1966, 1966–1997

Model	Handgrip Strength, kg						Trunk Muscle Endurance (*n* = 4,964)		
	Men (*n* = 2,505)			Women (*n* = 2,700)					
	β^a^	95% CI	*P* Value	β^a^	95% CI	*P* Value	HR^b^	95% CI	*P* Value
Model 1^c^	−0.3	−0.6, 0.1	0.15	0.03	−0.2, 0.3	0.78	0.93	0.90, 0.96	<0.001
Model 2^d^	−0.2	−0.5, 0.2	0.33	0.1	−0.2, 0.3	0.60	0.96	0.93, 0.99	0.02
Model 3^e^	−0.2	−0.5, 0.2	0.28	0.03	−0.2, 0.3	0.78	0.97	0.94, 1.00	0.03
Model 4^f^	−0.2	−0.5, 0.2	0.32	0.1	−0.2, 0.3	0.60	0.97	0.94, 0.99	0.02

Abbreviations: CI, confidence interval; HR, hazard ratio; LTL, leukocyte telomere length; SD, standard deviation.

^a^ Beta coefficients represent the average difference in handgrip strength per 1-SD increase in log_n_-transformed LTL.

^b^ HRs represent the difference in the chances of failing to complete the trunk muscle endurance test per 1-SD increase in log_n_-transformed LTL (lower HRs indicating higher trunk endurance).

^c^ Model 1—adjusted for age and LTL measurement batch (plus sex in the trunk muscle endurance analysis).

^d^ Model 2—adjusted for model 1 variables plus body mass index, socioeconomic position, diet quality, smoking status, and alcohol consumption.

^e^ Model 3—adjusted for model 2 variables plus C-reactive protein.

^f^ Model 4—adjusted for model 2 variables plus physical activity.

There was no evidence of effect modification by sex in LTL–aerobic fitness and LTL–trunk endurance associations (*P*’s for interactions with sex were 0.74 and 0.35, respectively). There was also no evidence that associations examined in linear regression models deviated from linearity (for nonlinear terms, all *P*’s ≥ 0.29) or that Cox hazard ratios deviated from proportionality across survival curves. Web Tables 2 and 3 show results from main-model analyses repeated after exclusion of extreme LTL outlier values (≥3 standard deviation). These reflected the same pattern of results as that seen in the main models for aerobic fitness and handgrip strength. The slight attenuation of the LTL–trunk muscle endurance association observed between model 1 and subsequent models in the main analyses was more pronounced in the outlier-exclusion analyses, particularly in models that adjusted for CRP and physical activity, which brought the results closer to the null. Finally, in models that mutually adjusted for the physical fitness measures (Web Tables 4 and 5), the LTL–aerobic fitness association was attenuated slightly after the inclusion of adjustments for handgrip strength and trunk muscle endurance. Similarly, the inclusion of adjustments for aerobic fitness and handgrip strength slightly attenuated the association of LTL with trunk muscle endurance in comparison with the confounder-adjusted model.

## DISCUSSION

### Findings

Our findings support the hypothesis that longer LTL is associated with higher aerobic fitness and trunk muscle endurance (but not with handgrip strength) in young adulthood. The slight attenuation of the association between aerobic fitness and LTL after adjustment for CRP suggests that inflammatory pathways may partly mediate the relationship between TL and cardiorespiratory fitness. Given that LTL was associated with trunk muscle endurance but not with handgrip strength, our findings suggest that TL may relate differently to varying aspects of muscular fitness in young adults.

The association that we observed between an objective measure of aerobic fitness and LTL is consistent with some previously reported correlations of LTL with maximal oxygen consumption in small numbers of endurance-trained athletes (13, 14), and also with a positive association of LTL with submaximal aerobic fitness in a cross-sectional survey of the US population (*n* = 1,764; mean age 34 years) (15). There are plausible mechanisms linking physical activity, aerobic fitness, and TL. Exercise increases aerobic fitness, while also increasing the cellular availability of nitric oxide (which protects cells from oxidative stress). In addition, exercise regimens appear to up-regulate expression of the telomerase reverse transcriptase gene (*TERT*) and other telomere-maintenance genes in mice (33), as well as in skeletal muscle of humans (34). Our finding that adjustment for CRP attenuated the association somewhat would also be consistent with underlying biology between TL and aerobic fitness, if long-term regular exercise suppressed low-grade inflammation—a condition hypothesized to shorten telomeres (35). Alternatively, given that higher adiposity is associated with increased inflammation, it is possible that the CRP adjustment resulted in the removal of some residual confounding by adiposity, if this was incompletely controlled for in the previous model (with fat mass being measured imprecisely by BMI).

Lack of association of LTL with handgrip strength in our data set is also consistent with most of the past studies investigating this trait in population-based samples of various ages (16–24). However, the observed association between LTL and trunk muscle endurance (and an association of LTL with isokinetic knee extensor strength, reported elsewhere (36)) could indicate a relationship between TL and muscle performance that is not well reflected by aspects of muscle function measured by handgrip strength or by other fitness parameters commonly measured in cohort studies of elderly subjects, such as walking speed or chair-rise tests. Experimental evidence suggests that there may be a role for TL in determining muscle function. Although skeletal muscle is a postmitotic tissue (where a lack of cell replication means there is no age-related telomere loss), its maintenance depends upon pools of progenitor satellite cells, which could be reduced in size with age if shortened telomeres trigger senescence/apoptosis (37). It is unclear whether telomere deterioration could produce meaningful proportions of critically short TL by young adulthood (as in our sample) to induce cellular senescence or apoptosis and, in turn, affect muscle function. Other potential mechanisms might explain how TL variation above critically short thresholds could affect muscle performance, such as complex interactions between telomeres, production of the p53 protein, and mitochondrial function (38). Thus, TL could affect muscular fitness directly and also have an impact on aerobic fitness by influencing the capacity for exercise. This would be particularly relevant in later life, when telomere dysfunction increases, muscle function declines sharply, and physical activity typically decreases (2). If this evidence has not arisen due to residual confounding or chance, such pathways could therefore be bidirectional.

Although physical activity is a determinant of physical fitness, adjustment for activity levels had little impact on the observed associations. Self-reported variation in activity between individuals is prone to measurement error (39), and more complete adjustment for variation may have attenuated the associations. However, measurement of physical activity records behavior, whereas physical fitness is an adaptive state determined by both heritable and environmental factors, some of which could also be related to LTL independently of activity (e.g., shared genetic components). It is worth noting that the association of LTL with trunk endurance was attenuated slightly after the small number of samples with outlier LTL measures was excluded from analyses. However, we were unable to determine whether extreme values represented true biological outliers (which could have helped produce a stronger association via more pronounced effects) or assay artifacts.

### Strengths and limitations

This study's main strengths were the use of a very large, nonselective, and homogeneous population sample and the possession of data on several objective measures of physical fitness. The principal limitation of this research was its cross-sectional design, which restricts inferences on causality or directions of associations. A second limitation is that TL was measured from leukocytes, rather than from cell types that would possibly be more relevant to our hypotheses (e.g., from skeletal muscle). However, measures of TL between tissues have been shown to correlate strongly, including to a high degree between leukocytes and skeletal muscle (40), and blood is the most practicable tissue to collect for measuring TL in large-scale epidemiologic settings.

### Conclusions

Our findings provide firm evidence that LTL is associated with measures of aerobic and muscular fitness in young adults. These associations may reflect biological pathways involving telomeres that underlie the age-related declines in aerobic and muscular fitness. Disparities between these findings and similar past studies could be attributable to differences in sample sizes, the types of physical fitness measures used, and/or methods of measurement (objective or subjective measures, maximal or submaximal tests). Further research is necessary to examine whether our results for aerobic fitness and muscle endurance can be replicated in other large studies with comparable, objective measures of physical fitness. Ideally, these studies would build upon our research by employing a longitudinal design, which could facilitate assessment of average changes in LTL and physical fitness in relation to each other over 2 or more time points and help with inference about the directions of associations. With the identification of genetic variants that are robustly associated with LTL, aerobic fitness, and muscle performance (already available for LTL (8)), it may be possible to conduct “Mendelian randomization” studies to assist with inferences about causality and resolve questions on the directions of associations. These studies could also help investigators assess the lifelong contribution of these traits to the development of inactivity, frailty, sarcopenia, and/or associated disease outcomes in old age (41). This would provide insights into the scope of possible hazard mitigation through improvements in TL and aerobic and muscular fitness within populations.

## Supplementary Material

Web Material
