# Supplementary material for: Associations of Leukocyte Telomere Length With Aerobic and Muscular Fitness in Young Adults
Source: Am J Epidemiol. 2017 Mar 1;185(7):529–37. doi: 10.1093/aje/kww123 (PMC5860435; doi:10.1093/aje/kww123)
Supplement: Web Material [file kww123williamswebmaterialfinal.pdf]

Web Table 1. Univariable trends in participant characteristics by fifths of the LTL distribution

	Lowest Fifth		2nd Fifth		3rd Fifth		4th Fifth		Highest Fifth		*P* Value**
	<0.87		0.87 to 1.05		>1.05 to 1.24		>1.24 to 1.51		>1.51		
Age (years)	31.18	(31.15, 31.20)	31.17	(31.15, 31.20)	31.14	(31.11, 31.17)	31.14	(31.12, 31.17)	31.12	(31.09, 31.15)	<0.001
Sex (% female)	47.3	(44.3, 50.3)	49.0	(46.0, 52.0)	52.2	(49.2, 55.2)	52.8	(49.8, 55.8)	57.4	(54.4, 60.3)	<0.001
BMI (kg/m^2)*	24.7	(24.4, 24.9)	24.6	(24.3, 24.8)	24.6	(24.4, 24.8)	24.0	(23.8, 24.3)	23.9	(23.7, 24.2)	<0.001
Socioeconomic position (%)											0.14
Farmer	2.7	(1.9, 3.9)	4.6	(3.5, 6.1)	3.8	(2.8, 5.1)	3.7	(2.7, 5.0)	4.0	(3.0, 5.3)	
Professional	25.2	(22.6, 27.9)	22.5	(20.1, 25.1)	25.5	(23.0, 28.3)	23.9	(21.5, 26.6)	22. 3	(19.8, 24.9)	
Skilled worker	29.6	(26.9, 32.4)	30.0	(27.3, 32.8)	31.5	(28.8, 34.4)	28.9	(26.3, 31.8)	33.5	(30.7, 36.4)	
Unskilled worker	27.7	(25.1, 30.5)	26.8	(24.2, 29.5)	24.2	(21.7, 26.9)	26.1	(23.6, 28.8)	23.5	(21, 26.1)	
Other	14.8	(12.7, 17)	16.1	(14, 18.4)	14.9	(12.9, 17.2)	17.3	(15.1, 19.7)	16.8	(14.6, 19.1)	
Physical activity (MET-hours/week)*	9.7	(9.0, 10.5)	9.7	(9.0, 10.5)	10.2	(9.4, 11.0)	9.8	(9.1, 10.6)	10.5	(9.7, 11.3)	0.23
Diet quality (% per category)											0.02
0	6.0	(4.7, 7.6)	6.4	(5.1, 8.1)	8.1	(6.6, 9.9)	9.2	(7.6, 11.1)	7.8	(6.3, 9.5)	
1	22.3	(19.9, 24.9)	22.6	(20.2, 25.2)	25.4	(22.9, 28.2)	24.8	(22.3, 27.5)	26.2	(23.7, 29)	
2	30.2	(27.5, 33)	31.7	(29, 34.6)	30.5	(27.8, 33.3)	28.9	(26.2, 31.7)	29.6	(27, 32.5)	
3	30.8	(28.1, 33.7)	26.9	(24.3, 29.6)	23.8	(21.4, 26.5)	25.2	(22.6, 27.9)	26.2	(23.7, 29)	
4 or 5	10.7	(9, 12.7)	12.4	(10.5, 14.5)	12.1	(10.3, 14.2)	12.0	(10.2, 14.1)	10.1	(8.5, 12.1)	
Ever smoker (%)	64.1	(61.2, 67.0)	66.1	(63.2, 68.9)	62.3	(59.4, 65.2)	63.0	(60.1, 65.9)	61.2	(58.2, 64.1)	0.06
Cigarette consumption per day (% non-smokers, or per tertile of use)											0.10
none	62.2	(59.2, 65)	62.1	(59.1, 64.9)	62.5	(59.6, 65.4)	65.2	(62.3, 68)	66.8	(63.9, 69.5)	
1st tertile	11.7	(9.9, 13.8)	11.7	(9.9, 13.8)	12.7	(10.8, 14.8)	13.5	(11.6, 15.7)	12.6	(10.7, 14.7)	
2nd tertile	14.9	(12.8, 17.1)	14.2	(12.2, 16.4)	13.8	(11.9, 16)	11.6	(9.8, 13.7)	12.1	(10.3, 14.2)	
3rd tertile	11.3	(9.5, 13.3)	12.0	(10.2, 14.1)	11.0	(9.2, 13)	9.7	(8, 11.6)	8.5	(7, 10.4)	

Alternate tobacco use (%)	4.6	(3.5, 6.1)	3.3	(2.4, 4.6)	3.0	(2.1, 4.2)	3.2	(2.3, 4.5)	2.7	(1.9, 3.9)	0.03
Alcohol consumption (g/day)*	4.6	(4.2, 5.1)	5.2	(4.7, 5.7)	4.5	(4.1, 5)	4.2	(3.8, 4.6)	3.8	(3.4, 4.2)	0.03
CRP (mg/L)*	0.87	(0.79, 0.95)	0.86	(0.78, 0.94)	0.82	(0.75, 0.9)	0.68	(0.62, 0.74)	0.66	(0.60, 0.73)	<0.001
Step test (BPM)	148.9	(147.7, 150.0)	148.9	(147.8, 150.1)	148.3	(147.2, 149.4)	147.5	(146.3, 148.6)	147.4	(146.2, 148.5)	0.002
Hand grip strength (kg)											
Male	49.8	(49.0, 50.7)	49.5	(48.7, 50.3)	50.2	(49.3, 51.0)	49.3	(48.5, 50.2)	48.9	(48.0, 49.8)	0.13
Female	28.0	(27.4, 28.6)	28.5	(27.9, 29)	28.0	(27.4, 28.5)	28.2	(27.6, 28.8)	28.1	(27.6, 28.7)	0.93
Trunk muscle endurance (seconds)*	153.0	(148.7, 157.3)	156.7	(152.5, 161.1)	161.7	(157.3, 166.2)	165.2	(160.7, 169.8)	162.3	(157.9, 166.8)	0.001

BMI: body mass index; BPM: beats per minute; CRP: C-reactive protein; MET: metabolic equivalent of task.

* Log_n-transformed variables used for tests of trends across fifths of LTL, due to non-normal distributions of original variables.

** *P* values are from linear or logistic regression across fifths of TS ratio, or χ^2 tests for categorical variables.

Web Table 2. Associations of LTL with aerobic fitness, excluding those with extreme LTL outlying values (*n* = 4,903)†

	**β***	**95% CI**	***P* Value**
Model 1	−2.3	−3.8, −0.9	0.002
Model 2	−1.7	−3.1, −0.3	0.02
Model 3	−1.6	−3.0, −0.2	0.03
Model 4	−1.7	−3.0, −0.3	0.02

† Participants with LTL values ≥3 SD from the mean of $\log_n$-transformed LTL distribution were excluded (*n* = 49).

* β's represent average difference in heart rate per one SD increase in mean relative LTL (lower heart rates indicating higher aerobic fitness).

Model 1—adjusted for age, sex and LTL batch.

Model 2—as model 1 plus BMI, SEP, physical activity, diet quality, smoking status, and alcohol consumption.

Model 3—as model 2 plus CRP.

Model 4—as model 2 plus physical activity.

Web Table 3. Associations of LTL with handgrip strength (stratified by sex) and trunk muscle endurance (not stratified), excluding those with extreme LTL outlying values†

	**Handgrip in Males (*n* = 2,482)**			**Handgrip in Females (*n* = 2,672)**			**Trunk Muscle Endurance (*n* = 4,916)**		
	**β***	**95% CI**	***P***	**β***	**95% CI**	***P***	**Hazard Ratio****	**95% CI**	***P***
Model 1	−0.3	−0.6, 0.1	0.15	0.1	−0.2, 0.3	0.61	0.94	0.90, 0.97	<0.001
Model 2	−0.2	−0.5, 0.2	0.32	0.1	−0.2, 0.3	0.47	0.97	0.94, 1.00	0.05
Model 3	−0.2	−0.5, 0.2	0.27	0.1	−0.2, 0.3	0.61	0.97	0.94, 1.00	0.08
Model 4	−0.2	−0.5, 0.2	0.31	0.1	−0.2, 0.3	0.47	0.97	0.94, 1.00	0.07

CI, confidence interval.

† Participants with LTL values ≥3SD from the mean of $\log_n$-transformed LTL distribution were excluded (*n* = 51 for handgrip analyses; *n* = 48 for trunk muscle endurance analyses).

* β's represent average difference in handgrip strength (kg) per one SD increase in $\log_n$-transformed LTL.

** Hazard ratios represent difference in the chances of failing to complete the trunk muscle endurance test per one SD increase in $\log_n$-transformed LTL (i.e. lower ratios are indicative of higher trunk muscle endurance).

Model 1—adjusted for age and LTL batch (plus sex in the trunk muscle endurance analysis).

Model 2—as model 1 plus BMI, SEP, physical activity, diet quality, smoking status, and alcohol consumption.

Model 3—as model 2 plus CRP.

Model 4—as model 2 plus physical activity.

Web Table 4. Associations of LTL with aerobic fitness in a subset of participants with information on all physical fitness measures (*n* = 4,733)

	β	95% CI	***P* Value**
Model 1	−0.8	−1.3, −0.3	0.002
Model 2	−0.6	−1.1, −0.1	0.02
Model 3	−0.5	−1.0, −0.02	0.04
Model 4	−0.5	−1.0, −0.04	0.04

CI, confidence interval.

* β's represent average difference in heart rate per per one SD increase in mean relative LTL (lower heart rates indicating higher aerobic fitness).

Model 1—adjusted for age, sex and LTL batch.

Model 2—as model 1 plus BMI, SEP, physical activity, diet quality, smoking status, and alcohol consumption.

Model 3—as model 2 plus CRP.

Model 4—as model 2 plus handgrip strength and trunk muscle endurance.

Web Table 5. Associations of LTL with trunk muscle endurance in a subset of participants with information on all physical performance measures (*n* = 4,733)

	Hazard Ratio*	95% CI	*P* Value
Model 1	0.93	0.90, 0.96	<0.001
Model 2	0.96	0.93, 0.99	0.01
Model 3	0.96	0.94, 0.99	0.02
Model 4	0.96	0.94, 0.99	0.02

CI, confidence interval.

* Hazard ratios represent difference in the chances of failing to complete the trunk muscle endurance test per one SD increase in $\log_n$-transformed LTL (i.e. lower ratios are indicative of higher trunk muscle endurance).

Model 1—adjusted for age, sex and LTL batch.

Model 2—as model 1 plus BMI, SEP, physical activity, diet quality, smoking status, and alcohol consumption.

Model 3—as model 2 plus CRP.

Model 4—as model 2 plus aerobic fitness and handgrip strength.
